# Effects of size, neighbors, and site condition on tree growth in a subtropical evergreen and deciduous broad‐leaved mixed forest, China

**DOI:** 10.1002/ece3.1665

**Published:** 2015-10-20

**Authors:** Xiulian Chi, Zhiyao Tang, Zongqiang Xie, Qiang Guo, Mi Zhang, Jielin Ge, Gaoming Xiong, Jingyun Fang

**Affiliations:** ^1^ Department of Ecology College of Urban and Environmental Sciences and Key Laboratory for Earth Surface Processes Peking University Beijing 100871 China; ^2^ State Key Laboratory of Vegetation and Environmental Change Institute of Botany Chinese Academy of Sciences No. 20 Nanxincun, Xiangshan Beijing 100093 China

**Keywords:** Canopy stature, leaf phenology, neighborhood competition index, phylogenetic distance, successional trait

## Abstract

Successful growth of a tree is the result of combined effects of biotic and abiotic factors. It is important to understand how biotic and abiotic factors affect changes in forest structure and dynamics under environmental fluctuations. In this study, we explored the effects of initial size [diameter at breast height (DBH)], neighborhood competition, and site condition on tree growth, based on a 3‐year monitoring of tree growth rate in a permanent plot (120 × 80 m) of montane *Fagus engleriana–Cyclobalanopsis multiervis* mixed forest on Mt. Shennongjia, China. We measured DBH increments every 6 months from October 2011 to October 2014 by field‐made dendrometers and calculated the mean annual growth rate over the 3 years for each individual tree. We also measured and calculated twelve soil properties and five topographic variables for 384 grids of 5 × 5 m. We defined two distance‐dependent neighborhood competition indices with and without considerations of phylogenetic relatedness between trees and tested for significant differences in growth rates among functional groups. On average, trees in this mixed montane forest grew 0.07 cm year^−1^ in DBH. Deciduous, canopy, and early‐successional species grew faster than evergreen, small‐statured, and late‐successional species, respectively. Growth rates increased with initial DBH, but were not significantly related to neighborhood competition and site condition for overall trees. Phylogenetic relatedness between trees did not influence the neighborhood competition. Different factors were found to influence tree growth rates of different functional groups: Initial DBH was the dominant factor for all tree groups; neighborhood competition within 5 m radius decreased growth rates of evergreen trees; and site condition tended to be more related to growth rates of fast‐growing trees (deciduous, canopy, pioneer, and early‐successional species) than the slow‐growing trees (evergreen, understory, and late‐successional species).

## Introduction

The rate of tree growth within a forest is the result of combined effects of biotic and abiotic factors (e.g., Stoll and Newbery 2005; Coomes and Allen 2007; Rapp et al. 2012; Stephenson et al. 2014). Major biotic factors that influence tree growth include intrinsic characters of trees, such as initial size or age (Coomes and Allen 2007; Stephenson et al. 2014), genotype (Boyden et al. 2008), and functional traits (Prior et al. 2004; Poorter et al. 2008; Chaturvedi et al. 2011), and extrinsic variables, such as neighborhood interaction (Stoll and Newbery 2005), herbivory (Whittaker and Warrington 1985), and fungal (Hagerberg et al. 2003) and bacterial relations (Leblanc et al. 2005). Major abiotic factors that influence tree growth include climate (Feeley et al. 2007; Toledo et al. 2011), light availability (Rüger et al. 2011; Dong et al. 2012), soil nutrient levels (Baribault et al. 2012), and site disturbances (Uriarte et al. 2004a). Exploring how tree growth responds to biotic and abiotic factors will provide important insights into how species differ in their life‐history strategies in terms of resource acquisition, defense against natural enemies, and allocation to reproduction (Baker et al. 2003a; Rüger et al. 2011). This may be also important for predicting potential changes in carbon stocks and biodiversity under environmental changes (Rüger et al. 2011).

Tree growth rates are highly variable among different species (Baker et al. 2003b; Sánchez‐Gómez et al. 2008). For instance, deciduous trees tend to grow faster than evergreen trees possibly because of their higher specific leaf area (SLA) (Cornelissen et al. 1996). Baker et al. (2003b) found that the deciduous *Celtis mildbraedii* grew faster than the evergreen *Strombosia glaucescens* in both semi‐deciduous and evergreen forests in Ghana. Even within the same genus, Sánchez‐Gómez et al. (2008) found that *Pinus pinaster* grew faster than *Pinus sylvestris* in central Spain. The different growth rates among species may be correlated with their canopy stature or successional traits. For example, large‐statured species tend to grow faster than small‐statured species, as a result of larger diameter growth rates and higher levels of crown illumination (Baker et al. 2003a; Poorter et al. 2005). Pioneer species tend to grow faster than late‐successional species because they have greater photosynthetic plasticity and a more enhanced growth response to irradiance (Strauss‐Debenedetti and Bazzaz 1991; Baker et al. 2003a).

Within a species, tree growth rates vary significantly among different stands because of the site conditions, including topography and soil nutrient availability (Kariuki et al. 2006; Baribault et al. 2012). Soil nitrogen (N) and phosphate (P) availability, both individually and in combination, affect plant productivity and other biological processes (Vitousek et al. 2010). In the tropics, tree growth rates are also correlated with soil base cations, such as calcium and potassium (Baribault et al. 2012). Even within the same stand, individual trees from the same species may vary in growth rate across different size classes. Differences in light conditions, the ability to competition with others, and vigor due to differences in initial size can all influence growth rates (Coomes and Allen 2007; Herault et al. 2011). The correlation between tree growth rate and initial size within a species may be sigmoid (Stoll et al. 1994), hump‐shaped (Herault et al. 2011), positive (Enquist et al. 1999), or negative (Rapp et al. 2012).

Another factor that influences tree growth is neighborhood interaction. Previous studies have proposed that asymmetric competition is an important process shaping tree growth in the forests (Canham et al. 2004; Stoll and Newbery 2005; Lebrija‐Trejos et al. 2014). Occurrence of both conspecific and heterospecific neighbors may reduce tree growth (Stoll and Newbery 2005; Lebrija‐Trejos et al. 2014). For example, the negative density‐dependent hypothesis proposes that individuals from the same species compete more strongly for the same limited resources than heterospecific individuals would do (Janzen 1970; Stoll and Newbery 2005; Lebrija‐Trejos et al. 2014). This hypothesis was extended to phylogenetic density‐dependent effects of hetero‐species competition, proposing that plant performance is largely reduced by the closely related species because species' niches tend to be phylogenetically conservative (Uriarte et al. 2004b; Webb et al. 2006; Lebrija‐Trejos et al. 2014). Thus, closely related species compete more strongly for similar resources and are more easily depressed by similar natural enemies (Janzen 1970; Lebrija‐Trejos et al. 2014).

Forest ecologists have extensively explored the patterns and determinants of tree growth (Coomes and Allen 2007; Gómez‐Aparicio et al. 2011; Baribault et al. 2012). Primarily, these studies have examined single‐species or limited multispecies forests (Coomes and Allen 2007; Gómez‐Aparicio et al. 2011; Baribault et al. 2012) and focused on a limited subset of species rather than all species within a community (Stoll and Newbery 2005; Coomes and Allen 2007). Therefore, these studies provided limited information on the effects of biotic and abiotic factors on the tree growth at the community level. Drivers of tree growth may differ among functional groups. For example, Baribault et al. (2012) found that the growth of nonlegume species from a lowland wet tropical forest was dependent on soil base cations and phosphorus (P), while the growth of legume species was independent of soil resources, suggesting that resource demands varied among functional groups.

In this study, we used a 3‐year measurement of tree growth in a subtropical montane *Fagus engleriana–Cyclobalanopsis multiervis* mixed forest on Mt. Shennongjia, China, to explore the effects of initial size, neighborhood competition, and site condition on tree growth. The high species richness and various functional groups in this evergreen and deciduous broad‐leaved mixed forest provide a unique opportunity to explore the potential drivers of tree growth among different functional groups. In this regard, our study addressed four important questions: (1) How do growth rates vary across functional groups? (2) How do the initial size, neighborhood competition, and site condition affect tree growth? (3) Do phylogenetic relatedness between trees influence their competitive interactions? and (4) Do the effects of initial size, neighborhood competition, and site condition on tree growth differ among functional groups?

## Methods

### Study site

**Figure 1:**
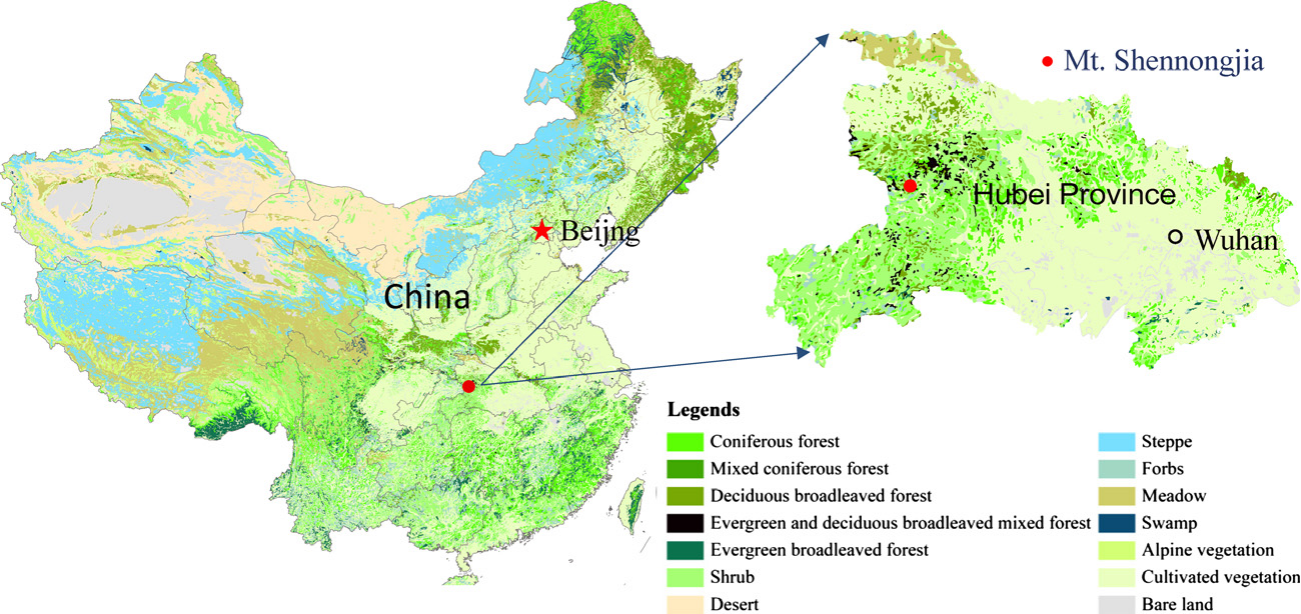
Map of China showing the location of the permanent plot on Mt. Shennongjia. The background was based on the “Vegetation Atlas of China (1:1,000,000)” (Editorial committee of Vegetation map of China, 2007).

The survey was conducted in a 120 × 80 m permanent forest plot established in 2001 at the elevation of 1650–1750 m on Mt. Shennongjia (31°19′4″N, 110°29′44″E, Fig. 1), the ecotone between subtropical evergreen broad‐leaved forest and temperate deciduous broad‐leaved forest in China (Ge et al. 2013). This area is characterized by a typical monsoon climate, with an annual precipitation of 1330 mm and annual mean temperature of 10.6°C. The soil type is montane yellow brown soil (Ge et al. 2013). The canopy layer is dominated by a deciduous broad‐leaved species *Fagus engleriana* and an evergreen broad‐leaved species *Cyclobalanopsis multiervis*. Density, mean diameter at breast height (DBH), and total stand basal area of the main stems of trees with DBH ≥ 5.0 cm are 1356 ha^−1^, 14.9 cm, and 31.85 m^2^ ha^−1^, respectively. The plot was divided into 384 grids of 5 × 5 m.

### Tree growth measurement

In October, 2011, we mapped and tagged 2261 free‐standing woody stems (all stems per tree) with DBH ≥ 5.0 cm, and installed field‐made steel dendrometer bands at ~1.3 m height (or about 30–50 cm above the top of any significant damage, i.e., fluting or deformation, or disease on these trees) (Baker et al. 2003b). The changes in windows of dendrometer bands (changes in tree circumference) were measured with digital calipers (precision of ± 0.01 mm) at approximately 6‐month intervals in April and October of each year from 2011 to 2014. Annual diameter increments were then calculated as the changes in circumference divided by *pi* and census interval (3 years in this study). In this analysis, only stems which were alive thorough the whole census interval were included. We also eliminated trees with annual diameter increments of more than 7.5 cm or with diameter shrinkage of more than 25% of their initial DBH (Rüger et al. 2011; Dong et al. 2012) and only focused on the main stems of multistem trees. In total, growth rates of 1302 main stems belonging to 78 species, 52 genera, and 33 families were included. Detailed information of these species can be found as Appendix S1.

### Topography and soil measurements

We used elevation, convexity, slope, and aspect to represent the topography of each grid. To do this, we first measured elevation of the four corners of this permanent plot using GPS as datum points. Secondly, we measured relative elevation at the four corners of each 5 × 5 m grid using the DQL‐1 forest compass (Harbin Optical Instrument Factory, Harbin, China) and calculated the real elevation according to the datum points. The mean elevation of the four corners was then calculated to represent the elevation of the grid. Convexity, slope and aspect were then calculated based on elevation of each grid according to Legendre et al. (2009). Convexity was calculated as the elevation of the focal grid minus the mean elevation of the eight surrounding grids, and the elevation of the center point minus the mean of the four corners for the edge grids (Legendre et al. 2009). Following Zar (1999), we further transformed the circular variable of aspect (in degrees) into two separate continuous variables, northness (northness = sin ((aspect in degrees × *pi*)/180)) and eastness (eastness = cos ((aspect in degrees × *pi*)/180)).

In August 2001, we sampled topsoil (0–10 cm depth) in the middle of each 20 × 20 m subplot (24 in total) and measured twelve soil properties referring to the standard methods of chemical analyses, namely pH, organic matter (SOM), concentration of total nitrogen (TN), total phosphorus (TP), NH_4_‐N, NO_3_‐N, K, Ca, Na, Mg, Fe, and Al. For details of soil sampling and measurements, please refer to Zhang (2005). We also carried out the semi‐variogram analysis for all soil properties, and found that the range parameters of all these models were larger than 20 m, which indicated that the sampling intensity could reflect significant space variation in soil properties (Appendix S2). We therefore interpolated these soil variables to grids of 5 × 5 m by Ordinary Kriging using GS+ 9.0 (GeoStatistics for the Environmental Sciences, by Gamma Design Software).

### Defining functional groups

In this study, we categorized the focal tree species into different functional groups according to their characteristics of leaf phenology, canopy stature, or successional traits. According to their leaf phenology, we grouped the species into evergreen (23 species, 635 individuals) and deciduous trees (55 species, 667 individuals). According to canopy stature, we categorized the species into treelets, understory, and canopy species based on the (nearly) largest DBH of individuals of the focal species (King et al. 2006). To do this, we first calculated the 95th percentile of DBH of all trees ≥0.1 × D_max_ (D95_0.1_) for species, where D_max_ is the largest DBH of each species. Species with 5 cm ≤ D95_0.1_ < 12 cm were defined as treelets species (20 species, 100 individuals), 12 cm ≤ D95_0.1_ < 25 cm as understory (24 species, 695 individuals), and D95_0.1_ ≥ 25 cm as canopy trees (34 species, 507 individuals) (King et al. 2006). For species with less than 10 individuals, we also referred to the records in “Flora of China” (http://www.floraofchina.org/) to get the potential height of them and define their canopy stature according to their potential heights. We found no difference between these two methods, which indicated there was no bias in adult stature estimation using the method of King et al. (2006). In addition, we categorized the tree species as pioneer (13 species, 67 individuals), early‐successional (33 species, 397 individuals), and late‐successional species (32 species, 838 individuals) according to Ge et al. (2013). Please refer to the Appendix S1 for detailed information on the functional groups of species.

### Data analysis

We applied principal component analysis (PCA) to reduce the covariation and possible redundancy of the 17 site condition variables (12 soil and five topographic variables). The first four axes retained 77.6% of the total variance (see Appendix S3). PCA axis 1 (PCA_1_) was mainly associated with soil properties including soil pH and concentrations of Ca, Mg, and TN. PCA axis 2 (PCA_2_) was mainly associated with topographic variables (northness, eastness, elevation, and slope), SOM, and NH_4_‐N concentration. PCA axis 3 (PCA_3_) was strongly correlated with slope, eastness, and K concentration; and PCA axis 4 (PCA_4_) was mainly contributed by concentrations of soil NO_3_‐N, Na, TP, and K.

The distance‐independent, semi‐distance‐independent, or distance‐dependent competition indices have been proposed to measure neighborhood effects (Sánchez‐Gómez et al. 2008). Here, we defined a neighborhood competition index (*NCI*) to represent the effects of neighboring trees within a defined radius on the focal tree by considering the relative size and spatial distance according to Canham et al. (2004):

(1) $${\text{NCI}}_{\mathrm{can}}=\frac{1}{{\text{DBH}}_{\mathrm{focal}}}\sum_{i=1}^{s}\sum_{j=1}^{n_{i}}\frac{{\text{DBH}}_{\mathrm{ij}}}{{\text{SD}}_{\mathrm{ij}}}$$

where NCI_can_ is the neighborhood competition index for the focal tree, DBH_focal_ is the DBH of the focal tree, *s* is the number of neighborhood species and *n*_i_ is the number of individuals of species *i* within a fixed neighborhood radius, DBH_ij_ is the DBH of the individual *j* of the species *i*, and SD_ij_ is the spatial distance (units: m) between the focal tree and competing tree *j* of species *i*.

However, NCI_can_ does not consider the effects of phylogenetic relatedness between the neighbors and focal trees. Therefore, we introduced the phylogenetic distance (PD) to define a phylogenetic distance‐weighted neighborhood competition index, NCI_pd_, to test whether the strength of competition between individual trees increases with their phylogenetic similarity. The NCI_pd_ was assumed to vary as an inverse function of the size of focal trees, the spatial and phylogenetic distance to the neighbors. The NCI_pd_ was calculated as:

(2) $${\text{NCI}}_{\mathrm{pd}}=\frac{1}{{\text{DBH}}_{\mathrm{focal}}}\sum_{i=1}^{s}\sum_{j=1}^{n_{i}}\frac{{\text{DBH}}_{\mathrm{ij}}}{{\text{SD}}_{\mathrm{ij}}*\left({\mathrm{PD}}_{\mathrm{ij}}+1\right)}$$

where PD_ij_ is the phylogenetic distance (units: Ma) of the focal tree and the competing tree *j* of species *i*. To avoid the effect of zero phylogenetic distance when the focal tree and the competitors were from the same species, all the PD_ij_ had 1 added when calculating the index.

To calculate phylogenetic distance (*PD*) among species, we first considered all 78 species in the plot as the community pool, and constructed a phylogenetic tree using Phylomatic (Webb and Donoghue 2005) based on the APG III topology. We then adjusted the branch length of phylogenetic tree to match the node age estimates reported by Wikström et al. (2001) using the bladj function within the software Phylocom 4.2 (Webb et al. 2008). Finally, we calculated the *PD* among all species with this software using the phydist function in the phylogeny with estimated branch length.

For trees with multiple stems, stems other than the main stem were defined to act as neighbors but were excluded from being focal trees. Neighboring trees were defined as those individuals located within 5 m or 10 m of a focal tree. To avoid edge effects, trees within 5 m or 10 m of the plot boundary were defined to act as neighbors but not as focal trees. We defined the neighborhood radius to be 5 m or 10 m to allow for sufficient sample sizes to be obtained for each functional group.

### Statistical analysis

We first compared the differences in growth rates among functional groups using paired *t*‐tests with Bonferroni correction with the data of 1302 main stems. We then applied a linear mixed model (LMM) to test the effects of initial DBH, NCI_can_ (or NCI_pd_), and site condition (PCA_1‐4_ of the topography and soil variables) on growth rates with 1022 and 776 main stems which were 5 m and 10 m inside the plot boundary, respectively. Initial DBH, NCI_can_ (or NCI_pd_), and PCA_1‐4_ were used as fixed effects. Grid‐specific random intercept was used to characterize autocorrelation in tree growth rate within the same grid, and species was included as a crossed random effect because tree growth rate of different species was expected to respond differently to other variables.

We constructed two models where only one type of NCI (NCI_can_ or NCI_pd_) was included as a fixed variable, and then compared with models' AICs to test the effects strength of PD on competition between trees. NCI_pd_ was not a better explanatory variable compared to NCI_can_ for most analyses (Table 1). Thus, we selected NCI_can_ as a neighborhood competition index to do the further analysis. We then compared the goodness of eight candidate linear mixed models: (1) M_0_: Null model only includes random effects; (2) M_S_: size model with initial DBH as fixed effect; (3) M_N_: neighborhood competition model with NCI_can_ as fixed effect; (4) M_H_: site condition model with site condition (PCA_1‐4_) as fixed effect; (5) M_SN_: size–neighborhood competition model with initial DBH and NCI_can_ as fixed effects; (6) M_SH_: size–site condition model with initial DBH and site condition (PCA_1‐4_) as fixed effects; (7) M_NH_: neighborhood competition–site condition model with NCI_can_ and site condition (PCA_1‐4_) as fixed effects; and (8) M_SNH_: full model with initial DBH, NCI_can_, and site condition (PCA_1‐4_) as fixed effects. Models with ΔAIC < 2 were considered equally valid (Burnham and Anderson 2002). We also calculated the marginal ($R_{\mathrm{mar}}^{2}$) and conditional *R* ^2^ ($R_{\mathrm{con}}^{2}$) of the models (Nakagawa and Schielzeth 2013). The $R_{\mathrm{mar}}^{2}$ represented the variance explained by fixed factors, whereas the $R_{\mathrm{con}}^{2}$ indicated variance explained by fixed and random factors.

**Table 1:** Model comparisons between two types of 5‐m‐ and 10‐m‐radius neighborhood competition index incorporating (NCI_pd_) and not incorporating (NCI_can_) phylogenetic distance between tree species

Functional group	5 m radius				10 m radius			
	Num	NCI_pd_	NCI_can_	ΔAIC	Num	NCI_pd_	NCI_can_	ΔAIC
Overall	1022	220.5	**206.3**	14.2	776	171.8	**148.2**	23.6
Evergreen	503	−258.2	−**264.5**	6.3	382	−225.4	−**229.8**	4.4
Deciduous	519	326.9	**319.1**	7.8	394	258.0	**240.1**	18.0
Canopy	410	297.5	**291.2**	6.3	325	225.9	**213.1**	12.7
Understory	538	−243.5	−**246.6**	3.1	400	−**174.3**	−**176.0**	**1.7**
Treelets	74	−**70.6**	−**71.2**	**0.6**	51	−42.7	−**45.0**	2.3
Pioneer	56	**47.4**	**48.6**	**1.2**	38	**33.9**	**35.0**	**1.1**
Early	290	169.0	**156.9**	12.1	221	141.3	**116.1**	25.2
Late	676	−32.0	−**38.4**	6.4	517	−36.9	−**45.1**	8.2

Num indicates the number of trees. ΔAIC was the differences between AICs of the two models (the best and the next) with different neighborhood competition indices. The (equally) most likely models are shown in bold.

All the continuous explanatory variables were first standardized and normalized before regressions; therefore, a positive coefficient of a factor in the model indicates a positive effect, and the larger the positive coefficient was, the stronger the positive effects of the factor. Initial DBH was directly log‐transformed (log (DBH)), while growth rate (GR) had 0.2 added and then log‐transformed (log (GR + 0.2)) before analysis to homogenize and normalize residuals of the models.

General linear mixed models were applied for a whole community, and for different leaf phenological groups (evergreen vs. deciduous), canopy stature groups (treelets vs. understory vs. canopy trees), and successional status groups (pioneer vs. early‐successional vs. late‐successional trees).

All analyses were carried out in R 3.0.3 (R Core Team, http://www.R-project.org/). The package “spatstat” was used to calculate topographic variables, “picante,” “splancs,” and “simba” combined to calculate the neighborhood competition index, “lmerTest” was used to carry out the analysis of general linear mixed models, and the package of “MuMIn” was applied to calculate the marginal and conditional *R* ^2^ of the mixed models.

## Results

### Statistics of tree growth rates

**Figure 2:**
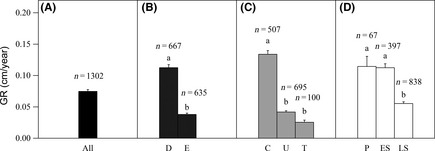
Annual growth rate (GR) of all species (A), species with different leaf phenology (B), canopy stature (C), and successional traits (D). *n* indicates the number of trees for each group. Different letters above the error bars indicate significant differences among groups at p_adj_. ≦ 0.05. D, deciduous; E, evergreen; C, canopy; U, understory; T, treelets; P, pioneer; ES, early‐successional; LS, late‐successional.

**Figure 3:**
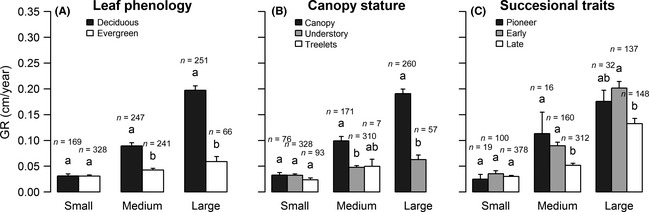
Annual growth rate (GR) for species with different (A) leaf phenology, (B) canopy stature, and (C) successional traits for different groups of size classes. Small, Medium, and Large indicate the initial diameter within the range of 5–10 cm, 10–20 cm, and ≥20 cm, respectively. n indicates the number of trees for each group. Different letters indicate differences, while similar letters indicate no differences, among groups within each size class significant at p_adj_. ≦ 0.05. Error bars represent the standard error.

On average, trees in the *Fagus engleriana–Cyclobalanopsis multiervi*s mixed forest of Mt. Shennongjia grew 0.07 cm (SD = 0.10 cm) annually (Fig. 2A). Deciduous species grew faster than evergreen species (0.11 cm year^−1^ and 0.04 cm year^−1^, respectively, *p*_adj_ < 0.001) (Fig. 2B); canopy species grew faster than understory and treelets species (0.13 cm year^−1^, 0.04 cm year^−1^, and 0.03 cm year^−1^, respectively, *p*_adj_ < 0.001) (Fig. 2C); and pioneer and early‐successional species grew faster than late‐successional species (0.11 cm year^−1^, 0.11 cm year^−1^, and 0.06 cm year^−1^, respectively, *p*_adj_ < 0.001) (Fig. 2D). To illustrate the differences of growth rate among different functional groups of trees within similar size, we grouped trees into different size classes according to their initial DBH. We found that for individuals within the same size classes, deciduous, canopy, and pioneer species still grew faster than evergreen, treelets, and late‐successional ones, respectively. Most of these differences were significant for the larger‐sized trees (size class 10 cm ≤ DBH < 20 cm and DBH ≥ 20 cm), but not for small‐sized trees (5 cm ≤ DBH < 10 cm; Fig. 3A–C).

### Influences of initial DBH, neighborhood competition, and site condition on tree growth rates

**Figure 4:**
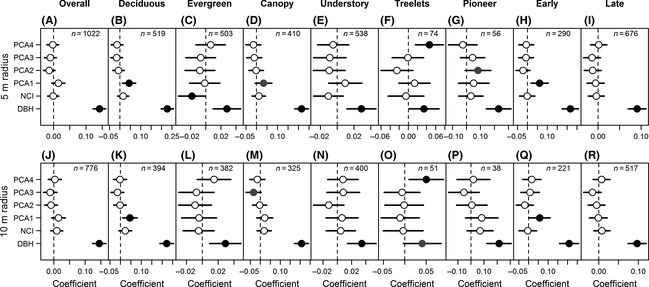
Factors included in the full models influencing annual growth rate (GR) of different datasets. DBH = initial DBH, NCI = NCI_can_, and PCA_1‐4_ indicate the first four axes of site condition variables. *n* indicates the number of trees for each group. Black and gray solid dots indicate significant (≦ 0.05) and marginally significant (0.05 < *P* ≦ 0.10) effects, respectively, while open dots indicate nonsignificant (*P* > 0.10) effects.

Considering competition within 5 m radius, for overall trees and trees from groups of canopy, pioneer, and late‐successional species, the best‐fit model of growth rates included only initial DBH as a fixed factor (M_S_); and an equally most likely model included both initial DBH and NCI_can_ (M_SN_) (Table 2). However, the initial DBH was the only significant factor positively related to growth rates (Fig. 4A, D, G and I). The fixed factors (initial DBH and NCI_can_) explained 11.7–33.8% of variance in growth rates of these groups of species (Table 2). We also found marginally significant relationships between PCA_1_ (Ca, Mg, TN, and pH) and growth rates of canopy trees (Fig. 4D), and between PCA_2_ (aspect, elevation, slope, SOM, and NH_4_‐N) and growth rates of pioneer trees, respectively (Fig. 4G). For evergreen and understory trees, M_SN_ was the best‐fit model (Table 2). Growth rates significantly increased with initial DBH for both groups of species, but only significantly decreased with NCI_can_ for groups of evergreen trees (Fig. 4C and E). Initial DBH and NCI_can_ together explained 3.8% and 2.5% of variance in growth rates of evergreen and understory trees, respectively (Table 2). For deciduous trees, treelets, and early‐successional trees, the best‐fit model included initial DBH and site condition as fixed factors (M_SH_), and the full model (M_SNH_) made an equally most likely model (Table 2). Growth rates increased with initial DBH for all tree groups (Fig. 4B, F and H), and were significantly correlated with PCA_1_ (pH, TN, Mg, and Ca) for deciduous and early‐successional trees (Fig. 4B and H), with PCA_4_ (NO_3_‐N, Na, TP, and K) for treelets. Initial DBH, neighborhood competition, and site condition together explained 22.1–38.7% of variance in growth rates of these three tree groups (Table 2).

**Table 2:** Goodness‐of‐fit in linear mixed models on tree growth rates based on the analysis with 5 m radius

Data type	Model fitness	M_0_	M_S_	M_N_	M_H_	M_SN_	M_SH_	M_NH_	M_SNH_
Overall	AIC	219.6	29.9	220.5	226.1	30.5	32.6	227.0	33.2
	ΔAIC	189.7	**0.0**	190.6	196.2	**0.6**	2.7	197.1	3.3
	$R_{\mathrm{mar}}^{2}$ (%)	0.0	21.1	0.1	0.1	21.2	21.6	0.2	21.8
	$R_{\mathrm{con}}^{2}$ (%)	43.0	45.0	43.0	42.7	45.0	45.1	42.7	45.2
Evergreen	AIC	−257.6	−269.2	−258.2	−250.7	−271.1	−262.5	−251.1	−264.2
	ΔAIC	13.5	**1.9**	12.9	20.4	**0.0**	8.6	20.0	6.9
	$R_{\mathrm{mar}}^{2}$ (%)	0.0	3.0	0.5	0.3	3.8	3.1	0.7	3.9
	$R_{\mathrm{con}}^{2}$ (%)	29.7	27.0	30.0	31.0	27.3	27.5	31.2	27.7
Deciduous	AIC	325.2	139.5	326.9	330.2	141.4	137.3	331.9	139.2
	ΔAIC	187.9	2.2	189.6	192.9	4.2	**0.0**	194.6	**1.9**
	$R_{\mathrm{mar}}^{2}$ (%)	0.0	35.7	0.1	0.6	35.7	37.3	0.7	37.3
	$R_{\mathrm{con}}^{2}$ (%)	30.5	50.3	30.1	29.7	50.4	51.6	29.4	51.7
Canopy	AIC	295.8	158.3	297.5	303.3	159.8	161.9	305.0	163.5
	ΔAIC	137.5	**0.0**	139.2	145.0	**1.5**	3.6	146.7	5.2
	$R_{\mathrm{mar}}^{2}$ (%)	0.0	29.9	0.1	0.2	29.9	30.8	0.3	30.7
	$R_{\mathrm{con}}^{2}$ (%)	24.6	53.9	24.3	23.8	54.1	54.8	23.6	55.0
Understory	AIC	−244.6	−254.8	−243.5	−238.5	−254.9	−249.7	−237.4	−250.0
	ΔAIC	10.3	**0.1**	11.4	16.4	**0.0**	5.2	17.5	5.0
	$R_{\mathrm{mar}}^{2}$ (%)	0.0	2.1	0.1	0.4	2.5	2.6	0.5	3.1
	$R_{\mathrm{con}}^{2}$ (%)	30.2	30.8	30.5	30.6	31.4	31.6	30.9	32.1
Treelets	AIC	−72.3	−75.3	−70.6	−75.8	−73.7	−78.8	−74.0	−77.1
	ΔAIC	6.5	3.5	8.2	3.0	5.1	**0.0**	4.9	**1.8**
	$R_{\mathrm{mar}}^{2}$ (%)	0.0	6.4	0.4	17.2	6.8	21.7	17.4	22.1
	$R_{\mathrm{con}}^{2}$ (%)	33.7	37.6	34.4	30.5	38.3	34.8	30.4	34.6
Pioneer	AIC	47.6	29.3	47.4	52.8	31.3	31.7	51.8	33.5
	ΔAIC	18.3	**0.0**	18.1	23.6	**2.0**	2.4	22.5	4.2
	$R_{\mathrm{mar}}^{2}$ (%)	0.0	33.9	4.2	6.3	33.8	39.5	12.5	39.4
	$R_{\mathrm{con}}^{2}$ (%)	72.6	69.8	77.9	73.8	70.1	73.1	79.5	74.9
Early	AIC	167.4	72.8	169.0	168.4	72.9	70.5	170.3	71.4
	ΔAIC	97.0	2.4	98.6	97.9	2.4	**0.0**	99.8	**0.9**
	$R_{\mathrm{mar}}^{2}$ (%)	0.0	34.7	0.2	2.6	35.8	38.0	2.6	38.7
	$R_{\mathrm{con}}^{2}$ (%)	38.1	54.2	38.5	34.6	56.2	52.5	34.8	54.0
Late	AIC	−32.6	−103.3	−32.0	−26.5	−102.2	−98.9	−25.7	−97.6
	ΔAIC	70.7	**0.0**	71.3	76.7	**1.1**	4.4	77.6	5.7
	$R_{\mathrm{mar}}^{2}$ (%)	0.0	11.8	0.2	0.3	11.7	12.0	0.4	11.9
	$R_{\mathrm{con}}^{2}$ (%)	43.5	39.0	43.7	43.9	39.0	38.6	44.1	38.6

M_0_: Null model only with random effects; M_S_: size model with initial DBH as fixed effect; M_N_: neighborhood competition model with NCI_can_ as fixed effect; M_H_: site condition model with site condition (PCA_1‐4_) as fixed effect; M_SN_: size–neighborhood competition model with initial DBH and NCI_can_ as fixed effects; M_SH_: size–site condition model with initial DBH and site condition (PCA_1‐4_) as fixed effects; M_NH_: neighborhood competition–site condition model with NCI_can_ and site condition (PCA_1‐4_) as fixed effects; and M_SNH_: full model with initial DBH, NCI_can_, and site condition as fixed effects. ΔAIC was calculated using AIC of each model minus the minimum AIC of all candidate models. The best‐fitting models are highlighted in bold.

When considering neighborhood competition within 10 m radius, M_S_ was the best‐fit model of growth rates for all tree groups except early‐successional species, with M_SN_ as an equally most likely model (see Appendix S4). For deciduous trees and treelets, M_SH_ and M_SNH_ were also the equally most likely models (Appendix S4). The fixed factors (initial DBH and NCI_can_) explained 2.5%~34.0% of variance in growth rates. Similar to the analysis with 5 m radius, the initial DBH was the only significant factor positively related to growth rates (Fig. 4J–R). Site condition was significantly (or marginally) related to growth rates for deciduous, canopy, treelets, and early‐successional trees (Fig. 4K, M, O and Q). However, NCI_can_ was not significantly related to growth rates of any tree group (Fig. 4J–R).

## Discussion

### Variations in tree growth rates among different functional groups

In this study, we found that growth rates varied significantly among functional groups (Figs. 2 and 3). Consistent with previous studies (Cornelissen et al. 1996, 1998), we found that deciduous trees grew faster than evergreen trees (Figs. 2B and 3A). One possible cause for this effect could be the higher specific leaf area (*SLA*) of deciduous trees (226.85 cm^2^ g^−1^) compared to evergreen trees (111.34 cm^2^ g^−1^) in this community (unpublished data, Cornelissen et al. 1996, 1998; Antúnez et al. 2001). *SLA* represents allocation of leaf biomass to light capture (Poorter et al. 2008). Higher *SLA* is frequently reported to be related to higher nutrient concentrations and mass‐based photosynthetic rates (Reich et al. 1992; Poorter et al. 2008); therefore, it may result in faster growth (Chaturvedi et al. 2011). Given the positive relationship between tree growth rate and initial DBH, another possible reason may stem from the larger initial DBH of deciduous trees than the evergreen trees (means of 18.0 cm and 11.6 cm).

Among groups differing in canopy stature, we found that tree growth rates were greater with greater adult size of species, that is, canopy species grew faster than understory and treelets species (Figs. 2C and 3B), consistent with growth rates in tropical rain forests (Thomas 1996). There may be several reasons for such a pattern. First, tree height tends to be higher in canopy species, than in understory and treelets species of the same size or during adulthood (Kohyama et al. 2003; Poorter et al. 2003). Greater heights would result in increases in mean crown illumination for canopy trees and, therefore, increases in their absolute growth rates (King et al. 2006). However, taller trees were also found to have lower light use efficiency; the trade‐off between higher light interception efficiency and lower light use efficiency would result in similar relative growth rates for trees of different canopy stature (Onoda et al. 2014). Second, larger‐statured species (i.e., canopy spp. in this study) have greater leaf‐level photosynthetic capacity than smaller‐statured species, even for individuals from different species within the same genus (Thomas and Bazzaz 1999), which could account for the former's higher intrinsic growth rates (Herault et al. 2011; Rüger et al. 2012). Third, the ages of individuals of the same size may be different, such that the larger‐statured species may be younger than the small‐statured species and the growth rates of small‐statured species (adults) would therefore be slower (King et al. 2006).

Among species differing in successional status, pioneer and early‐successional species grew faster than late‐successional species (Figs. 2D and 3C). Two possible reasons may exist for this pattern. First, at a given irradiance, pioneer species have higher intrinsic growth rates than more shade‐tolerant species, and are necessarily present in sites of high light availability, for example, gaps (Baker et al. 2003a; Poorter et al. 2008). Second, species of earlier stages of succession have greater photosynthetic plasticity and growth responses to irradiance (Shukla and Ramakrishnan 1986; Strauss‐Debenedetti and Bazzaz 1991) and nutrients (Fetcher et al. 1996) in comparison with shade‐tolerant species. The nonsignificant difference we found between pioneer and early‐successional trees may be partly due to their similar requirements and responses to light.

### Drivers of tree growth rates among functional groups

For all analyses, initial DBH and neighborhood competition index (NCI_can_) were included in the fullest, most likely models (Table 2; Appendix S4), indicating that initial size and neighborhood competition contributed to tree growth in this mixed forest. However, the neighborhood effect was only significant for evergreen trees within 5 m radius, and the importance of site condition to growth rates varied among functional groups.

Growth rates of overall trees and of different functional groups were greater with the greater initial DBH (linear relationship on log–log scale) (Fig. 4A–I). Within a species, larger sizes have two opposite effects on tree growth. On the one hand, larger trees tend to be more competitive and grow faster because they can reach higher light availability (King et al. 2006). On the other hand, larger trees may grow more slowly, as they may need to increase allocations to reproduction (Thomas 1996) and root and stem respiration (Ryan and Yoder 1997) because of reduced vigor (as a result of aging) (Herault et al. 2011). The balance between these two conflicting processes shapes the final size–growth relationships of a species, a functional group, or finally a forest community.

Although insignificant (except for evergreen trees), neighborhood competition index (*NCI*_*can*_) played a not‐inconsiderable role in tree growth as it was retained during the model selection processes (Table 2). Consistent with previous studies (Uriarte et al. 2004a; Stoll and Newbery 2005), we found negative effects of neighborhood competition (NCI_can_) on tree growth for most of our datasets (overall trees and evergreen, understory, treelets, and early‐ and late‐successional species). The occurrence of neighbors may enhance the resource limitation due to the asymmetric competition between the focal and neighboring trees, thus reducing growth rates to different degrees, dependent on the tolerance of competition of different species (Sánchez‐Gómez et al. 2008). In addition, it is widely reported that plant performance is largely reduced by closely related species because of their common resource requirements and natural enemies under the assumption of phylogenetically conservatism (Janzen 1970; Uriarte et al. 2004b; Webb et al. 2006; Lebrija‐Trejos et al. 2014). However, this is not supported in our study, as we found equal or even poorer model fitness when including phylogenetic distance in neighborhood competition index (Table 1). These results, to some extent, demonstrate the small effect of phylogenetic relatedness on competitive interactions between trees. Our result was consistent with the studies of Uriarte et al. (2010) and Kunstler et al. (2012), who found that competitive interactions between trees were not driven by phylogenetic similarity but rather by species' trait hierarchies. Furthermore, the low intensity of neighborhood effects may be caused by the limited neighborhood radius (5 m and 10 m) we used in this study. A radius of 5 m or 10 m may not capture enough competitive effects of neighbors, especially considering that different radii played a significant role for different functional groups and species in Borneo (Stoll and Newbery 2005). Further comparisons with different neighborhood radii in the future may improve our understanding of the contribution of neighborhood competition to tree growth.

Compared to initial size and neighborhood interaction, site condition played less important roles in growth rates of trees in this montane evergreen and deciduous broad‐leaved mixed forest. Specifically, we found that growth rates of deciduous trees, treelets, and early‐successional species were more strongly correlated with site condition than other groups (Table 2; Fig. 4B, F, H, K, O and Q). Growth rates of deciduous and early‐successional species were positively related to PCA_1_, which was mainly related to soil pH and concentrations of Mg Ca, and TN, while growth rates of treelets were positively related to PCA_4_, which was mainly contributed by concentrations of NO_3_‐N, Na, TP, and K (Appendix S3). These together indicated that nutrient availability, especially soil nitrogen and base cations, may influence tree growth rates of certain functional groups in this mixed forest. Interestingly, we also found that tree growth rates of canopy and pioneer species showed a marginally significant correlation with site condition variables (Fig. 4D, G and M). We therefore suggest that the fast‐growing species, such as trees from deciduous, canopy, pioneer, and early‐successional species (Figs. 2, 3, 4; Table 2), tended to be more resource‐limited (Finzi 2009; Baribault et al. 2012), because more nutrient resources were required to support the faster growth. The short‐lived leaves of the deciduous trees limited their growth period to the growing season, while the extended leaf longevity of evergreen trees enhances their nutrient use efficiency and/or long‐term carbon gain (Reich et al. 1992). Such limitations of nutrient availability also applied to the pioneer and early‐successional species (Huante et al. 1995; Fetcher et al. 1996).

In addition, we found that the fixed factors, including initial DBH, neighborhood competition index, and site condition, accounted for more variance of growth rates in deciduous, canopy, treelets, pioneer, and early‐successional trees compared with growth rates of other groups. These results, to some extent, suggested that other unconsidered factors, such as functional traits (Poorter et al. 2008; Chaturvedi et al. 2011), herbivory (Whittaker and Warrington 1985), and climate (Feeley et al. 2007; Toledo et al. 2011), may limit the growth rates of evergreen, understory, and late‐successional species. Moreover, some biases might be caused by not including the dead trees as the result of a biased sample in some populations. However, because only 67 trees died during the measurement interval (2011–2014), accounted for less than 3% of the total individuals at the beginning, we think these biases can be neglected in our study.

We also noticed that the combined effects of initial size, neighborhood competition, and/or site condition only explained a small fraction of variance in tree growth rates (2.9–39.5%). The random factors (species and spatial location of trees) explained more variances (6.7–35.0%) of tree growth of some functional groups (e.g., evergreen and understory) than the fixed variables (initial size, neighborhood competition index, and site condition), which indicated the important effects of species identity and spatial locations on tree growth in this evergreen and deciduous mixed forest. However, a large amount of variance remains unexplained, possibly because other unconsidered factors, for example, intraspecific genetic variability (Boyden et al. 2008), forest structure (Coomes et al. 2014), herbivory (Whittaker and Warrington 1985), soil water availability (Baker et al. 2003b), soil temperature (Landhäusser et al. 1996), or irradiance (Dong et al. 2012), may limit tree growth. Incorporating these factors in a future model with more individuals of each species may improve our understanding of tree growth under the scenario of climate change.

In summary, we explored the relative effects of initial size, neighborhood competition, and site condition on tree growth rates in a montane *Fagus engleriana–Cyclobalanopsis multiervis* mixed forest in China using general linear mixed models. We found that trees within this forest grew 0.07 cm year^−1^ in DBH. Trees from functional groups of deciduous, canopy, and early‐successional species grew faster than evergreen, understory, and late‐successional species. In general, tree growth rate was significantly influenced by initial size, but less by neighborhood competition and site condition (topography and soil characteristics). We also found that the different combinations of initial size, neighborhood competition, and site condition influenced tree growth rate among different functional groups. Specifically, initial size and neighborhood competition played more important roles than site condition. Furthermore, site condition was a more important factor to fast‐growing trees (deciduous, canopy, pioneer, and early‐successional species) than to slow‐growing trees (evergreen, understory, and late‐successional species). We concluded that besides the effects of species identity and spatial location of trees, initial size was generally a more important factor, suggesting that ontogenetic effects may shift growth pattern. The relative importance of neighborhood competition and site condition to tree growth in this montane evergreen and deciduous broad‐leaved mixed forest differed among different function groups of trees.

## Conflict of Interest

None declared.

## Supporting information


**Appendix S1.** Information of species included in this study.
**Appendix S2.** Variogram model fit parameters for soil properties in this study.
**Appendix S3.** Results of a principal component analysis based on the correlation matrix between topographic and soil variables.
**Appendix S4.** Goodness‐of‐fit in linear mixed models on tree growth rates based on the analysis 10 m radius.
