# Supplementary material for: Effects of size, neighbors, and site condition on tree growth in a subtropical evergreen and deciduous broad‐leaved mixed forest, China
Source: Ecol Evol. 2015 Oct 20;5(22):5149–61. doi: 10.1002/ece3.1665 (PMC6102529; doi:10.1002/ece3.1665)
Supplement: Supplementary file 1 — Appendix S1. Information of species included in this study. Appendix S2. Variogram model fit parameters for soil properties in this study. Appendix S3. Results of a principal component analysis based on the correlation matrix between topographic and soil variables. Appendix S4. Goodness‐of‐fit in linear mixed models on tree growth rates based on the analysis 10 m radius. [file ECE3-5-5149-s001.docx]

**Appendix S1.** Information of species included in this study

| Family | *Species* | LP | ST | CS | Num | mDBH | mGR | m*NCIcan* | m*NCIpd* | BA% | BA Rank |
| --- | --- | --- | --- | --- | --- | --- | --- | --- | --- | --- | --- |
| Aceraceae | *Acer davidii* Franch. | D | P | C | 10 | 16.4 | 0.13 | 8.36 | 0.56 | 0.83 | 29 |
| Aceraceae | *Acer flabellatum* Rehd. | D | P | C | 13 | 16.8 | 0.06 | 9.57 | 3.58 | 1.14 | 24 |
| Aceraceae | *Acer griseum* (Franch.) Pax | D | LS | C | 32 | 18.5 | 0.15 | 10.36 | 3.78 | 3.21 | 8 |
| Aceraceae | *Acer henryi* Pax | D | ES | U | 5 | 11.1 | 0.10 | 10.75 | 2.15 | 0.16 | 51 |
| Aceraceae | *Acer pictum* Thunb. | D | ES | C | 55 | 18.0 | 0.11 | 8.98 | 1.64 | 5.22 | 4 |
| Aceraceae | *Acer sinense* Pax | D | ES | U | 17 | 13.0 | 0.11 | 13.18 | 4.54 | 0.90 | 28 |
| Anacardiaceae | *Toxicodendron vernicifluum* (Stokes) F. A. Barkl. | D | P | C | 4 | 21.8 | 0.38 | 2.33 | 0.01 | 0.49 | 36 |
| Aquifoliaceae | *Ilex pedunculosa* Miq. | E | LS | U | 11 | 7.9 | 0.03 | 11.88 | 0.78 | 0.19 | 49 |
| Aquifoliaceae | *Ilex pernyi* Franch. | E | LS | T | 25 | 6.6 | 0.04 | 19.65 | 1.92 | 0.29 | 43 |
| Betulaceae | *Betula albosinensis* Burk. | D | P | C | 12 | 22.7 | 0.12 | 7.89 | 0.62 | 2.09 | 14 |
| Betulaceae | *Betula fargesii* Franch. | D | ES | C | 5 | 21.4 | 0.07 | 9.79 | 2.30 | 0.69 | 34 |
| Betulaceae | *Betula insignis* Franch. | D | ES | C | 14 | 22.2 | 0.14 | 5.49 | 2.50 | 2.13 | 13 |
| Betulaceae | *Betula luminifera* H. Winkl. | D | P | U | 2 | 13.5 | 0.03 | 4.57 | 0.01 | 0.10 | 60 |
| Betulaceae | *Carpinus fargesiana* H. Winkl. | D | ES | C | 16 | 18.6 | 0.13 | 9.59 | 3.27 | 1.75 | 17 |
| Betulaceae | *Corylus chinensis* Franch. | D | LS | C | 11 | 24.7 | 0.10 | 2.96 | 0.22 | 1.87 | 16 |
| Betulaceae | *Ostrya japonica* Sarg. | D | ES | C | 1 | 29.2 | 0.29 | 3.72 | 0.01 | 0.22 | 47 |
| Caprifoliaceae | *Viburnum rhytidophyllum* Hemsl. | E | ES | T | 1 | 5.5 | 0.01 | 16.84 | 0.04 | 0.01 | 78 |
| Caprifoliaceae | *Viburnum sympodiale* Graebn. | D | LS | T | 2 | 6.2 | 0.00 | 26.13 | 0.06 | 0.02 | 70 |
| Caprifoliaceae | *Weigela japonica* Thunb. | D | P | T | 2 | 9.4 | -0.01 | 5.57 | 0.01 | 0.04 | 66 |
| Celastraceae | *Euonymus hamiltonianus* Wall. | D | ES | T | 2 | 6.6 | 0.02 | 12.88 | 0.03 | 0.02 | 69 |
| Cercidiphyllaceae | *Cercidiphyllum japonicum* Sieb. & Zucc. | D | LS | C | 1 | 41.4 | 0.43 | 5.50 | 5.00 | 0.44 | 37 |
| Cornaceae | *Cornus controversa* Hemsl. | D | ES | C | 10 | 21.9 | 0.20 | 4.57 | 0.20 | 1.61 | 19 |
| Cornaceae | *Cornus kousa subsp. chinensis* (Osborn) Q. Y. Xiang | D | ES | U | 50 | 10.5 | 0.05 | 19.39 | 9.62 | 1.58 | 20 |
| Cornaceae | *Cornus ulotricha* C. K. Schneid. et Wangerin | D | ES | C | 25 | 16.7 | 0.09 | 7.03 | 0.97 | 2.20 | 12 |
| Daphniphyllaceae | *Daphniphyllum macropodum* Miq. | E | LS | U | 1 | 19.3 | 0.16 | 2.55 | 0.01 | 0.10 | 61 |
| Ebeceae | *Diospyros lotus* L. | D | P | C | 3 | 27.6 | 0.14 | 9.28 | 5.77 | 0.59 | 35 |
| Elaeagceae | *Elaeagnus lanceolata* Warb. | E | P | T | 2 | 6.2 | 0.00 | 22.24 | 0.06 | 0.02 | 71 |
| Ericaceae | *Lyonia ovalifolia var. lanceolata* (Wall.) Hand.-Mazz. | D | LS | U | 12 | 10.3 | 0.00 | 24.58 | 5.39 | 0.36 | 41 |
| Ericaceae | *Pieris formosa* (Wall.) D. Don | E | LS | U | 3 | 13.0 | 0.01 | 17.85 | 5.40 | 0.13 | 53 |
| Ericaceae | *Rhododendron concinnum* Hemsl. | E | LS | T | 1 | 7.3 | 0.01 | 2.63 | 0.02 | 0.01 | 74 |
| Ericaceae | *Rhododendron hypoglaucum* Hemsl. | E | LS | U | 130 | 9.1 | 0.02 | 18.66 | 6.45 | 3.08 | 10 |
| Ericaceae | *Rhododendron simsii* Planch. | D | ES | T | 1 | 7.3 | 0.00 | 22.97 | 8.80 | 0.01 | 75 |
| Eupteleaceae | *Euptelea pleiosperma* Hook. f. et Thom. | D | ES | U | 14 | 13.3 | 0.08 | 9.01 | 2.97 | 0.73 | 33 |
| Fabaceae | *Cladrastis delavayi* (Franch.) Prain | D | LS | C | 3 | 16.9 | 0.08 | 13.45 | 0.05 | 0.24 | 45 |
| Fagaceae | *Castanea henryi (*Skan) Rehd. et Wils. | D | ES | C | 7 | 34.6 | 0.27 | 5.69 | 2.29 | 2.27 | 11 |
| Fagaceae | *Cyclobalanopsis multinervis* Cheng et T. Hong | E | LS | U | 353 | 12.1 | 0.04 | 14.82 | 7.15 | 15.85 | 1 |
| Fagaceae | *Cyclobalanopsis oxyodon* (Miq.) Oerst*.* | E | LS | U | 7 | 12.6 | 0.05 | 12.79 | 2.16 | 0.39 | 39 |
| Fagaceae | *Fagus engleriana* Seem. | D | LS | C | 89 | 20.3 | 0.14 | 20.70 | 17.06 | 11.41 | 2 |
| Fagaceae | *Lithocarpus cleistocarpus* (Seem.) Rehd. et Wils. | E | LS | C | 29 | 21.4 | 0.06 | 6.42 | 2.64 | 3.81 | 6 |
| Fagaceae | *Lithocarpus glaber* (Thunb.) kai | E | LS | T | 1 | 10.5 | 0.06 | 8.92 | 0.03 | 0.03 | 68 |
| Fagaceae | *Lithocarpus henryi* (Seem.) Rehd. et Wils. | E | LS | C | 15 | 19.2 | 0.07 | 11.88 | 2.88 | 1.87 | 15 |
| Fagaceae | *Quercus aliena var. acutiserrata* Maxim. ex Wenz*.* | D | ES | C | 7 | 41.8 | 0.25 | 2.40 | 0.10 | 3.53 | 7 |
| Fagaceae | *Quercus engleriana* Seem. | E | LS | U | 20 | 12.8 | 0.02 | 16.47 | 4.06 | 1.05 | 26 |
| Fagaceae | *Quercus serrata* Thunb. | D | ES | C | 17 | 26.3 | 0.16 | 5.32 | 0.44 | 3.20 | 9 |
| Fagaceae | *Quercus spinosa* David. ex Franch. | E | LS | U | 3 | 7.7 | 0.02 | 14.62 | 0.06 | 0.05 | 65 |
| Flacourtiaceae | *Idesia polycarpa* Maxim. | D | P | C | 1 | 36.1 | 0.11 | 0.75 | 0.00 | 0.33 | 42 |
| Hamamelidaceae | *Corylopsis platypetala* Rehd. et Wils. | D | LS | T | 18 | 6.9 | 0.02 | 15.98 | 5.78 | 0.23 | 46 |
| Hydrangeaceae | *Philadelphus sericanthus* Koehne | D | ES | T | 5 | 6.5 | 0.00 | 13.89 | 2.25 | 0.06 | 63 |
| Juglandaceae | *Cyclocarya paliurus* (Batal.) Iljinsk. | D | LS | C | 7 | 19.1 | 0.09 | 10.27 | 1.35 | 0.78 | 32 |
| Juglandaceae | *Juglans mandshurica* Maxim. | D | ES | U | 1 | 21.1 | 0.26 | 3.26 | 0.01 | 0.11 | 57 |
| Juglandaceae | *Platycarya strobilacea* Sieb.et Zucc. | D | P | C | 10 | 21.7 | 0.21 | 6.82 | 1.71 | 1.49 | 21 |
| Lauraceae | *Lindera fragrans* Oliv. | E | LS | T | 3 | 8.1 | 0.00 | 7.38 | 0.02 | 0.05 | 64 |
| Lauraceae | *Lindera megaphylla* Hemsl. | E | LS | C | 3 | 21.5 | 0.22 | 4.86 | 0.67 | 0.37 | 40 |
| Lauraceae | *Lindera obtusiloba* Bl. | D | ES | U | 30 | 13.5 | 0.09 | 9.85 | 1.41 | 1.63 | 18 |
| Lauraceae | *Lindera pulcherrima var. hemsleyana* (Diels) H. P. Tsui | E | LS | T | 1 | 7.4 | -0.01 | 11.10 | 0.03 | 0.01 | 73 |
| Lauraceae | *Litsea ichangensis* Gamble | D | ES | T | 1 | 8.1 | 0.06 | 7.91 | 0.02 | 0.02 | 72 |
| Lauraceae | *Sassafras tzumu* (Hemsl.) Hemsl. | D | P | C | 1 | 31.8 | 0.12 | 2.99 | 0.01 | 0.26 | 44 |
| Magnoliaceae | *Yulania sprengeri* (Pamp.) D. L. Fu | D | ES | C | 9 | 17.4 | 0.08 | 5.98 | 1.21 | 0.78 | 31 |
| Meliaceae | *Toona sinensis* (A. Juss.) Roem. | D | ES | C | 31 | 22.9 | 0.30 | 4.95 | 1.72 | 5.00 | 5 |
| Moraceae | *Ficus heteromorpha* Hemsl. | D | P | T | 1 | 7.1 | 0.02 | 3.69 | 0.01 | 0.01 | 76 |
| Nyssaceae | *Davidia involucrata* Baill. | D | LS | U | 4 | 18.6 | 0.14 | 4.03 | 0.01 | 0.40 | 38 |
| Oleaceae | *Fraxinus insularis* Hemsl. | D | ES | U | 1 | 13.1 | 0.12 | 1.53 | 0.00 | 0.04 | 67 |
| Oleaceae | *Osmanthus armatus* Diels | E | LS | T | 10 | 7.1 | 0.03 | 18.97 | 0.14 | 0.13 | 54 |
| Rosaceae | *Cerasus clarofolia* (Schneid.) Yü et Li | D | ES | U | 5 | 11.1 | 0.09 | 10.51 | 0.03 | 0.20 | 48 |
| Rosaceae | *Photinia beauverdiana* Schneid. | D | ES | U | 1 | 22.9 | 0.01 | 4.18 | 0.01 | 0.13 | 52 |
| Rosaceae | *Sorbus alnifolia* (Sieb. et Zucc.) K. Koch | D | ES | C | 8 | 19.9 | 0.11 | 10.63 | 4.95 | 0.98 | 27 |
| Rosaceae | *Sorbus aronioides* Rehd. | D | ES | U | 4 | 12.9 | 0.02 | 20.39 | 16.19 | 0.17 | 50 |
| Rosaceae | *Sorbus folgneri* (Schneid.) Rehd. | D | ES | C | 15 | 15.4 | 0.15 | 11.06 | 2.51 | 1.08 | 25 |
| Rosaceae | *Sorbus zahlbruckneri* Schneid. | D | ES | U | 1 | 16.4 | 0.00 | 5.34 | 0.02 | 0.07 | 62 |
| Sabiaceae | *Meliosma flexuosa* Pamp. | D | ES | T | 10 | 6.4 | 0.02 | 10.94 | 1.08 | 0.11 | 59 |
| Salicaceae | *Salix wallichiana* Anderss. | D | P | C | 6 | 22.5 | 0.05 | 5.08 | 1.67 | 0.82 | 30 |
| Styracaceae | *Styrax hemsleyanus* Diels | D | LS | U | 18 | 16.0 | 0.04 | 7.05 | 1.47 | 1.34 | 22 |
| Symplocaceae | *Symplocos lucida* (Thunb.) Siebold et Zucc. | E | LS | T | 7 | 7.5 | 0.06 | 16.06 | 0.49 | 0.11 | 58 |
| Taxaceae | *Taxus wallichiana var. chinensis* (Pilg.) Florin | E | LS | T | 6 | 8.9 | 0.03 | 22.69 | 7.77 | 0.13 | 56 |
| Taxaceae | *Torreya fargesii* Franch*.* | E | LS | T | 1 | 5.9 | -0.01 | 18.73 | 0.03 | 0.01 | 77 |
| Theaceae | *Eurya hebeclados* Ling | E | ES | U | 2 | 14.5 | 0.05 | 10.67 | 6.47 | 0.13 | 55 |
| Theaceae | *Stewartia sinensis* Rehd. et E. H. Wilson | D | LS | C | 11 | 19.1 | 0.15 | 8.82 | 2.37 | 1.21 | 23 |
| Tiliaceae | *Tilia oliveri* Szyszyl. | D | ES | C | 26 | 25.5 | 0.09 | 8.13 | 2.00 | 5.83 | 3 |

Note: LP, leaf phenology, D and E represent deciduous and evergreen species, respectively; ST, successional traits, P, ES and LS represent pioneer, early- and late-successional species, respectively; CS, canopy status, C, U and T represent canopy, understory and treelets species, respectively; Num, number of observations; mDBH, mean diameter at breast height (units: cm); mGR, mean absolute diameter increment (units: cm.yr^-1^); m*NCIcan*, mean neighborhood index cited from Canham *et al.* (2004) within 5 m radius; m*NCIpd*, mean phylogeny adjusted neighborhood index within 5 m radius; BA%, the proportion of basal area to total basal area in the plot (units: %); BA Rank, the order of proportion, indicating the relative dominance of the species in the plot.

**Appendix S2.** Variogram model fit parameters for soil properties in this study. Effective range for the exponential variogram model is estimated at three-fold the fitted range, because the variogram approaches the sill only asymptotically.

| Soil variable | Fitted Model | Nugget | Sill | Effective range (m) | R^2^ |
| --- | --- | --- | --- | --- | --- |
| pH | Spherical model | 0.17 | 1.708 | 151.7 | 0.989 |
| Organic matter | Spherical model | 2.14 | 9.181 | 66.7 | 1 |
| Total nitrogen | Spherical model | 0.00171 | 0.01401 | 151.8 | 0.989 |
| Total phosphorus | Linear model | 0.01604 | 0.02212 | 65.05 | 0.273 |
| NH_4_-N | Exponential model | 5.7 | 50.17 | 42.6 | 0.998 |
| NO_3_-N | Linear model | 2.42425 | 2.42425 | 65.05 | 0.892 |
| K | Exponential model | 0.0001 | 0.0644 | 46.2 | 0.899 |
| Ca | Spherical model | 0.243 | 2.318 | 210.9 | 0.888 |
| Na | Spherical model | 0.0118 | 0.1036 | 54.6 | 1 |
| Mg | Spherical model | 0.0336 | 0.1672 | 179.8 | 0.654 |
| Al | Spherical model | 1.73 | 5.195 | 146.4 | 0.987 |
| Fe | Spherical model | 0.444 | 1.472 | 100.5 | 0.999 |

**Appendix S3.** Results of a principal component analysis based on the correlation matrix between topographic and soil variables

|  | PCA_1_ | PCA_2_ | PCA_3_ | PCA_4_ |
| --- | --- | --- | --- | --- |
| Standard deviation | 2.63 | 1.68 | 1.45 | 1.16 |
| Proportion of Variance（%） | 40.68 | 16.56 | 12.43 | 7.92 |
| Cumulative Proportion（%） | 40.68 | 57.24 | 69.67 | 77.59 |
| Eigenvectors |  |  |  |  |
| Elevation | -0.26 | -0.35 | -0.13 | 0.15 |
| Convexity | -0.28 | -0.24 |  |  |
| Slope |  | -0.31 | 0.50 |  |
| Eastness |  | -0.35 | 0.46 |  |
| Northness |  | 0.36 | -0.17 |  |
| pH | 0.32 | 0.16 | 0.18 |  |
| Organic matter | 0.26 | -0.31 | -0.23 |  |
| Total nitrogen | 0.34 | -0.17 |  | -0.11 |
| Total phosphorus | 0.24 | -0.19 | -0.25 | 0.36 |
| NH_4_-N | 0.17 | -0.37 | -0.14 | -0.26 |
| NO_3_-N | 0.22 | -0.13 | -0.23 | 0.53 |
| K |  | 0.19 | 0.49 | 0.32 |
| Ca | 0.36 |  |  | 0.15 |
| Na | -0.19 | 0.15 |  | 0.46 |
| Mg | 0.34 |  |  | 0.15 |
| Al | -0.29 | -0.21 |  | 0.25 |
| Fe | -0.25 | -0.18 |  | 0.23 |

**Appendix S4.** Goodness-of-fit in linear mixed models on tree growth rates based on the analysis 10 m radius . M_0_: null model only with random effects; M_S_: size model with initial DBH as fixed effects; M_N_: neighborhood competition model with *NCI_can_* as fixed effects; M_H_: site condition model with site condition (PCA_1-4_) as fixed effects; M_SN_: size- neighborhood competition model with initial DBH and *NCI_can_* as fixed effects; M_SH_: size-site condiction model with initial DBH and site condition (PCA_1-4_) as fixed effects; M_NH_: neighborhood competition-site condition model with *NCI_can_* and site condition (PCA_1-4_) as fixed effects; M_SNH_: full model with initial DBH, *NCI_can_* and site condition as fixed effects. △AIC was calculated using AIC of each model minus the minimum AIC of all candidate models. The best-fitting models are highlighted in bold.

| Data type | Model fitness | M_0_ | M_S_ | M_N_ | M_H_ | M_SN_ | M_SH_ | M_NH_ | M_SNH_ |
| --- | --- | --- | --- | --- | --- | --- | --- | --- | --- |
| Overall | AIC | 170.2 | 13.6 | 171.8 | 176.7 | 15.6 | 18.1 | 178.4 | 20.1 |
|  | △AIC | 156.6 | **0.0** | 158.2 | 163.2 | **2.0** | 4.5 | 164.8 | 6.5 |
|  | R^2^_mar_ (%) | 0.0 | 21.7 | 0.1 | 0.2 | 21.7 | 22.1 | 0.2 | 22.1 |
|  | R^2^_con_ (%) | 38.3 | 42.9 | 38.2 | 38.4 | 42.9 | 43.6 | 38.4 | 43.6 |
| Evergreen | AIC | -226.4 | -234.8 | -225.4 | -222.0 | -233.9 | -230.1 | -220.8 | -229.1 |
|  | △AIC | 8.4 | **0.0** | 9.5 | 12.8 | **1.0** | 4.7 | 14.0 | 5.8 |
|  | R^2^_mar_ (%) | 0.0 | 2.8 | 0.3 | 1.3 | 3.1 | 3.8 | 1.5 | 4.0 |
|  | R^2^_con_ (%) | 21.1 | 23.4 | 21.1 | 22.7 | 23.2 | 24.5 | 22.6 | 24.3 |
| Deciduous | AIC | 256.2 | 114.1 | 258.0 | 263.1 | 115.5 | 114.5 | 264.9 | 115.8 |
|  | △AIC | 142.1 | **0.0** | 143.9 | 149.0 | **1.4** | **0.4** | 150.8 | **1.7** |
|  | R^2^_mar_ (%) | 0.0 | 33.9 | 0.1 | 0.3 | 34.0 | 35.2 | 0.4 | 35.3 |
|  | R^2^_con_ (%) | 17.1 | 43.0 | 16.7 | 16.6 | 43.1 | 44.9 | 16.4 | 45.1 |
| Canopy | AIC | 224.1 | 117.7 | 225.9 | 227.7 | 118.5 | 120.1 | 229.6 | 120.8 |
|  | △AIC | 106.3 | **0.0** | 108.1 | 110.0 | **0.8** | 2.4 | 111.8 | 3.1 |
|  | R^2^_mar_ (%) | 0.0 | 29.4 | 0.1 | 1.7 | 29.4 | 30.9 | 1.8 | 30.9 |
|  | R^2^_con_ (%) | 17.3 | 48.0 | 17.0 | 16.1 | 48.2 | 49.0 | 15.9 | 48.8 |
| Understory | AIC | -176.2 | -185.0 | -174.3 | -170.6 | -183.4 | -179.8 | -168.8 | -178.1 |
|  | △AIC | 8.8 | **0.0** | 10.7 | 14.4 | **1.7** | 5.2 | 16.3 | 6.9 |
|  | R^2^_mar_ (%) | 0.0 | 2.3 | 0.0 | 0.6 | 2.5 | 3.1 | 0.6 | 3.2 |
|  | R^2^_con_ (%) | 35.7 | 34.6 | 35.7 | 34.5 | 34.4 | 33.9 | 34.5 | 33.7 |
| Treelets | AIC | -44.4 | -47.3 | -42.7 | -44.3 | -46.2 | -46.6 | -43.2 | -45.8 |
|  | △AIC | 3.0 | **0.0** | 4.7 | 3.1 | **1.1** | **0.7** | 4.2 | **1.6** |
|  | R^2^_mar_ (%) | 0.0 | 9.0 | 0.8 | 17.6 | 10.4 | 23.4 | 19.6 | 25.9 |
|  | R^2^_con_ (%) | 29.5 | 37.4 | 31.6 | 20.8 | 39.7 | 30.2 | 22.0 | 32.6 |
| Pioneer | AIC | 34.9 | 20.5 | 33.9 | 40.5 | 22.3 | 25.6 | 37.8 | 27.0 |
|  | △AIC | 14.5 | **0.0** | 13.4 | 20.0 | **1.9** | 5.1 | 17.3 | 6.5 |
|  | R^2^_mar_ (%) | 0.0 | 31.7 | 9.0 | 7.1 | 31.2 | 35.9 | 21.5 | 36.2 |
|  | R^2^_con_ (%) | 20.1 | 62.9 | 44.0 | 30.5 | 62.5 | 63.9 | 56.7 | 66.0 |
| Early | AIC | 140.3 | 72.3 | 141.3 | 144.8 | 71.3 | 74.8 | 145.8 | 73.9 |
|  | △AIC | 69.0 | **1.0** | 70.0 | 73.5 | **0.0** | 3.4 | 74.5 | 2.6 |
|  | R^2^_mar_ (%) | 0.0 | 31.9 | 0.6 | 1.7 | 33.2 | 33.9 | 2.3 | 35.2 |
|  | R^2^_con_ (%) | 14.8 | 47.5 | 19.1 | 14.5 | 52.1 | 47.6 | 13.9 | 51.8 |
| Late | AIC | -38.6 | -103.1 | -36.9 | -34.2 | -101.2 | -98.0 | -32.4 | -96.1 |
|  | △AIC | 64.5 | **0.0** | 66.2 | 69.0 | **2.0** | 5.1 | 70.7 | 7.0 |
|  | R^2^_mar_ (%) | 0.0 | 13.7 | 0.0 | 0.7 | 13.8 | 14.0 | 0.7 | 14.1 |
|  | R^2^_con_ (%) | 41.5 | 37.7 | 41.6 | 42.6 | 37.7 | 37.9 | 42.6 | 37.9 |
